# Genome-wide mapping of chromatin marks from 1,000 cells to study epigenetic reprogramming in primordial germ cells

**DOI:** 10.1186/1756-8935-6-S1-P9

**Published:** 2013-03-18

**Authors:** Julie Brind’Amour, Sheng Liu, Mehdi Karimi, Aaron Bogutz, Matthew Lorincz

**Affiliations:** 1 Department of Medical Genetics, University of British Columbia, Vancouver, British Columbia, Canada, V6T1Z3

## 

Germline development is characterized by genome-wide reprogramming of DNA methylation. Recent work has enlightened the dynamics of DNA methylation in primordial germ cells (PGCs), but knowledge of histone modification dynamics at these developmental stages remains limited, mostly due to the difficulty in obtaining enough high quality chromatin immunoprecipitation (ChIP) material for sequencing. Previous work in our laboratory has demonstrated the importance of histone methyltransferases in silencing retroelements [1] and a subset of germline-specific genes [2] in embryonic stem cells. Here, we sought to develop a reliable ChIP-sequencing protocol to study the dynamics of histone modification during the DNA methylation reprogramming that occurs in PGCs.

We have developed a scaled down native ChIP and sequencing library construction protocol that can be performed on small cell numbers. We optimized sample fragmentation, antibody concentration, ChIP conditions, library construction and amplification to generate high quality, high resolution H3K9me3 sequencing libraries from as little as 1,000 embryonic stem cells. Paired-end sequencing (Illumina HiSeq) of these pooled and indexed libraries generated an average of 28 million aligned read pairs (with 6 libraries per sequencing lane), with under 20% duplicate reads, for an average of 23 million unique read pairs. Under optimized conditions, we found that over 85% of the peaks identified using standard native ChIP-sequencing (using 2 million cells as starting material) were also detected by our small cell number native ChIP-seq protocol. We also found excellent reproducibility between independent ChIP experiments. Using this optimized small cell number native ChIP-seq protocol, we generated genome-wide H3 and H3K9me3 profiles from 1,000 E13.5 PGCs and identified a unique cohort of genes and retroelements enriched for this repressive mark. Integration of this chip-seq data with DNA methylation/WGBS and transcriptome data generated at the same developmental stage will be presented.
